# Aging Population, Balanced Diet and China’s Grain Demand

**DOI:** 10.3390/nu15132877

**Published:** 2023-06-25

**Authors:** Xiuli Liu, Mun S. Ho, Geoffrey J. D. Hewings, Yuxing Dou, Shouyang Wang, Guangzhou Wang, Dabo Guan, Shantong Li

**Affiliations:** 1 Academy of Mathematics and Systems Science, Chinese Academy of Sciences, Beijing 100190, China; 15229933168@163.com (Y.D.); sywang@amss.ac.cn (S.W.); 2 University of Chinese Academy of Sciences, Beijing 100049, China; 3 Center for Forecasting Science, Chinese Academy of Sciences, Beijing 100190, China; 4 Harvard China Project on Energy, Economy and Environment, School of Engineering and Applied Sciences, Harvard University, Cambridge, MA 02138, USA; munho@seas.harvard.edu; 5 Regional Economics Applications Laboratory, University of Illinois at Urbana-Champaign, Urbana, IL 61801, USA; hewings@illinois.edu; 6 Institute of Population and Labor Economics, Chinese Academy of Social Sciences, Beijing 100006, China; wangguangzhou@126.com; 7 Department of Earth System Science, Tsinghua University, Beijing 100084, China; guandabo@tsinghua.edu; 8 Development Research Center of the State Council, Beijing 100010, China; shantong@drc.gov.cn

**Keywords:** grain demand, dietary structure, aging population, urbanization, food waste, COVID-19

## Abstract

The need to make more accurate grain demand (GD) forecasting has become a major topic in the current international grain security discussion. Our research aims to improve short-term GD prediction by establishing a multi-factor model that integrates the key factors: shifts in dietary structures, population size and age structure, urbanization, food waste, and the impact of COVID-19. These factors were not considered simultaneously in previous research. To illustrate the model, we projected China’s annual GDP from 2022 to 2025. We calibrated key parameters such as conversion coefficients from animal foods to feed grain, standard person consumption ratios, and population size using the latest surveys and statistical data that were either out of date or missing in previous research. Results indicate that if the change in diets continued at the rate as observed during 2013–2019 (scenario 1), China’s GD is projected to be 629.35 million tons in 2022 and 658.16 million tons in 2025. However, if diets shift to align with the recommendations in the Dietary Guideline for Chinese Residents 2022 (scenario 2), GD would be lower by 5.9–11.1% annually compared to scenario 1. A reduction in feed grain accounts for 68% of this change. Furthermore, for every 1 percentage point increase in the population adopting a balanced diet, GD would fall by 0.44–0.73 million tons annually during that period. Overlooking changes in the population age structure could lead to an overprediction of annual GDP by 3.8% from 2022 to 2025. With an aging population, China’s GD would fall slightly, and adopting a balanced diet would not lead to an increase in GD but would have positive impacts on human health and the environment. Our sensitivity analysis indicated that reducing food waste, particularly cereal, livestock, and poultry waste, would have significant effects on reducing GD, offsetting the higher demand due to rising urbanization and higher incomes. These results underscore the significance of simultaneous consideration of multiple factors, particularly the dietary structure and demographic composition, resulting in a more accurate prediction of GD. Our findings should be useful for policymakers concerning grain security, health, and environmental protection.

## 1. Introduction

**Figure 1:**
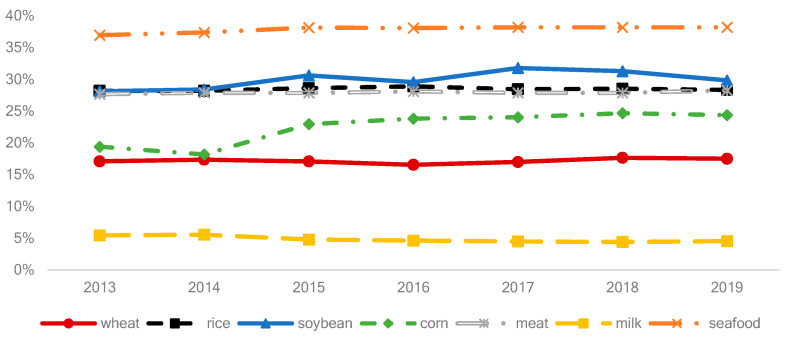
The proportion of China’s food consumption in the world’s food consumption by weight. Data source: FAOSTAT.

Sustainably meeting global grain demand is a critical challenge facing humanity and has attracted significant attention from researchers and policymakers [1,2,3], and recent work has discussed the additional difficulties during the COVID-19 pandemic [4,5,6]. Food security, as defined by the UN’s Sustainable Development Goals (Target 2), means ensuring sufficient supplies of food and nutrients, and grain plays a crucial role in meeting these needs. Global and regional grain demand (GD) had changed significantly in the past half-century due to rapid economic growth, population increase, rural-urban migrations, aging populations, and shifts in dietary structures [7,8]. Economic development in some populous countries, particularly the diet transformation, is expected to increase GD, thereby placing enormous pressure on grain supplies within these countries and globally [9,10]. China, as the world’s most populous country and the largest food consumer, has a significant impact on global GD, with its soybean, rice, and meat consumption each accounting for approximately 30% of the world’s total consumption by weight during 2013–2019, and seafood accounting for 38% (see Figure 1). The dominance of China in global food production and consumption has been noted by many, including [11,12], especially since the fifth version of the Dietary Guidelines for Chinese Residents (DGCR 2022) was published by the government in 2022. It is likely that the diets of many Chinese people will change towards DGCR 2022 [13,14,15]. If so, there is a need to emphasize the role of changes in dietary structure when predicting China’s GD.

There were various projections of future GD in China (Table S1) and around the world, and they varied widely; the projected global GD growth from 2005 to 2050 ranges from 35 percent to 110 percent [5]. Even the near-term projections for China’s GD in 2020, varied from 480 to 741 million tons (Table S1). This uncertainty in projections has made formulating appropriate grain supply plans challenging for policymakers [16,17]. It is important to make more accurate GD forecasting to provide a basis for policymaking in grain production, consumption and trade to ensure grain security, a topic that has become a major international issue.

During the past few decades, a wide range of models and data sources have been applied to GD prediction (e.g., [18,19,20]). The research methods can be roughly classified into three categories [16]. The first category is “complex mechanistic” models, which are often built on interacting economic, ecological, demographic, and/or climate sub-models [21,22,23,24]. The second category uses a simple mechanistic approach to predict demand based on a simple (Engel’s Law) relationship between income and kilocalorie consumption [18,25,26,27,28,29]. The third kind, phenomenological models, assume that current trends of increasing kilocalorie consumption will continue [20,30,31,32]. Different modeling methods had resulted in a variety of forecasts for global or regional GD. However, [16] found that these differences were not due to the complexity of the models. The purely phenomenological (time-trend) model predictions fell within the same range as simple and complex mechanistic models. While complex models are flexible and include expected changes in agricultural output, food prices, and trade, they are opaque and sometimes irreproducible. Simpler models are easier to interpret and reproduce but may not be as accurate. Therefore, decisions about model complexity should be made based on the research or policy questions of interest when models have comparable forecasting accuracy.

Given this range of approaches, several studies have analyzed the reasons behind varying predictions of GD—different assumed parameters, such as conversion coefficients from animal foods to feed grain (Table S2), price elasticities, food waste rate, etc. [10,33]. Other sources of variation are the projected population, age structure, and especially the dietary structure [20]. Uncertainties in the growth of meat and dairy consumption in South and East Asia, particularly China and India, were the primary sources of uncertainty in global GD forecasts [34]. These problems also exist when projecting China’s GD. Only a handful of China GD projections, such as [19,35], have emphasized the role of changes in dietary structure. However, they failed to consider food waste and the potential impacts of the COVID-19 pandemic, which had not occurred at the time of their research. Integrating the main impact factors of GD and recalibrating key parameters using the latest information would improve the projections on GD [1,5,34].

We aim to improve short-term prediction of GD by integrating these main drivers of GD, especially dietary structure and demographic structure. To do this, we established a multi-factor model that simultaneously integrates population size, age structure, dietary structure, urbanization, food waste rate, and the impacts of COVID-19. Our model is in the complex mechanistic model category. When we set the values of some parameters in the model, we refer to phenomenological models, assuming that their current trends will continue in the short term. We project China’s GD under three scenarios. In scenario 1 (S1), we assume that the dietary structure would evolve towards the balanced diet guidelines in DGCR 2022 at the annual average pace during 2013–2019, adjusting for the impacts of COVID-19. The results in scenario 1 provide a prediction of China’s annual GD from 2022 to 2025, assuming no special efforts are made to shift diets or reduce food waste during this period. In scenario 2 (S2), we assume that all residents will adopt the DGCR 2022 guidelines during 2022–2025. In scenario 3 (S3), we project GD, again assuming adoption of DGCR 2022 guidelines but ignoring the effect of changing age structure. Comparing these 3 scenarios gives the impact of changing diets and population age structure on China’s future annual GD. We then conduct a sensitivity analysis to assess the impact of different coefficients, such as the ratio of people adopting DGCR 2022, food waste, urbanization rate, etc., on the reduction of GD. Such sensitivity analyses tell us the effects of changes in these factors on reducing GD, thus providing guidance on effective strategies to reduce GD.

We make two contributions to the GD literature. First, we make short-term GD predictions using a “complex mechanistic” model, integrating the main driving factors of population size and its age structure, dietary structure shifts, urbanization, food waste, and the impacts of COVID-19. We updated or calibrated key parameters in the model, such as the standard person consumption ratios, conversion coefficients from animal foods to feed grain, and population size, with the latest survey and statistical data, which were either out of date or missed in previous studies. Secondly, by simulating different scenarios, we show the impacts of different factors on GD, especially aging and shifts in dietary structures. The results identify potential ways to reduce GD in China and should be useful for the government to formulate grain supply plans and policies to effectively reduce GD and at the same time promote healthy diets.

The remainder of the paper is as follows: the second section introduces the research methodology. The third section is the model application in China, and Section 4 presents the results. We discuss the results, model limitations, and their implications in Section 5. The last section draws conclusions.

## 2. Research Methodology

**Figure 2:**
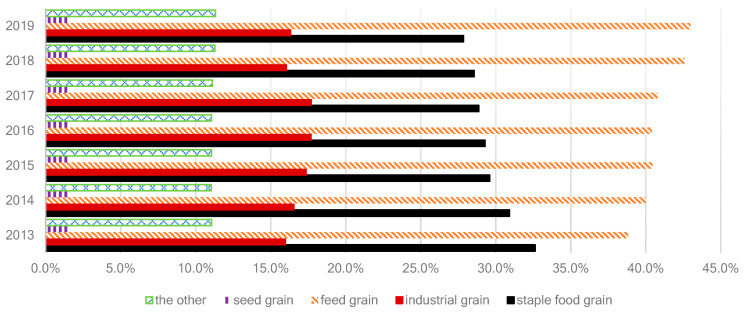
The annual consumption structure of grain by weight in China. Data source: FAOSTAT.

Based on the classification standards of Food and Agriculture Organization of the United Nations (FAO), GD can be divided into staple food grain, feed grain, industrial grain, seed and other grain, as shown in Figure 2. Due to the diversity of grain uses, we employed a functional decomposition analysis to first decompose food grain demand into staple food grain (denoted *c* = 1) and feed grain (*c* = 2). We refer to the sum of the two as food grain and is represented by *c* = 3. The demand for food grain is subject to greater uncertainty than other types of grain, which is the main driver of fluctuations in GD, as noted by previous studies [36,37]. Consequently, we emphasize the calculation of food grain demand in the model. We considered three age groups–0–14, 15–64, 65+—that were used in our food survey, and indexed them by *i* = 1, 2, 3. The types of food consumed (the diet) were represented by *j* = {Cereal, tubers and beans, Livestock and poultry meat, Aquatic products, …} as listed in Table 1. The demand for feed grain was calculated based on the conversion rate calculation method. Since the proportion of the other types of grain in China was stable during 2013–2019, we consider the other types of grain by defining a lower bound and an upper bound on their historical change trend, following the approach in [30,38].

We establish a multi-factor model of GD prediction, incorporating the major drivers—shifts in dietary structure, changes in population size, age structure, urbanization, and food waste rates. Since we are projecting for the near term 2022–2025, we also integrate the effect of COVID-19. There are other important factors that are known to impact GD, in particular, food prices and income. We do not explicitly include these effects but represent them via changes in diets (changes in the types of food consumed). Refs. [10,39,40] showed that dietary preferences were mostly determined by prices and household incomes, and our explicit treatment of dietary structures gave an indirect treatment of these factors.

*Step 1. Modeling consumption per capita and ‘standard persons’*

Let $f_{ij}^{t}$ denote the quantity of food type *j* consumed on average by a person in age group *i* in year *t*, and $t_{jc}^{t}$ denote the coefficient transferring food type *j* to grain requirement of type *c*. $t_{jc}^{t}$ is calculated with Equations (s1)–(s4) in the Supplementary Materials. Equation (1) gives the amount of grain *c* consumed by a group *i* person in year *t* ($gd_{ic}^{t}$) as the sum over all the types of grain requirements transferred to food *j*:

(1) $$gd_{ic}^{t}=\sum_{j}f_{ij}^{t}*t_{jc}^{t}\;(i=1,\;2,\;3;\;j=1,\;2\ldots )$$

**Figure 3:**
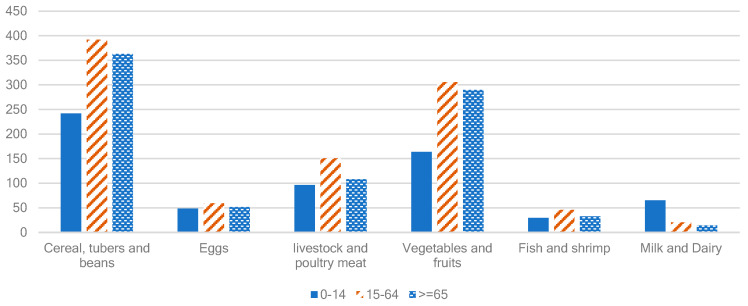
The dietary pattern of three age groups in China in 2017 (g/per capita per day).

Much of the literature used the concept of a standard person and expressing the requirement of a person of age *i* relative to a standard age group. According to FAOSTAT, a person aged 17–18 years old had the highest energy demand, which then falls with age. On average, the energy demand of a 60–69-year-old person is 70.3% of the peak value by age, while an 80–89-year-old person has a ratio of 49.9% [41]. For the food consumption by differently aged persons in China, a national survey conducted by the University of Chinese Academy of Sciences (UCAS) in 2017 [42] shows that the volume of food consumption was the highest for those aged 15–64 for all types of food except for milk and dairy products (see Figure 3).

We, therefore, define the standard person consumption ratio for type *c* grain by persons in age group *i*, $spcc_{ic}$, as the ratio of their average grain demand to the demand by age group *i* = 2 (15–64 years old), giving a simple account for the impact of age structure on GD:

(2) $$spcc_{ic}=gd_{ic}^{t}/gd_{2c}^{t}\;(i=1,\;2,\;3;\;c=1,\;2,\;3)$$

We define the number of standard persons in age group *i*, gender *k*, in year *t*, $sp_{ik}^{t}$, as:

(3) $$sp_{ik}^{t}=p_{ik}^{t}*spcc_{i3}\;k=\{1\;(\mathrm{male}),\;2\;(\mathrm{female})\}$$

where $p_{ik}^{t}$ is the population of age group *i*, gender *k* in year *t*.

*Step 2. Calculating annual food grain demand*

In Equation (4), we write the actual per capita gross demand for type *c* grain by a gender *k* person in urban areas in year *t* ($agu_{ck}^{t})\;$as the grain embodied in the sum over all food types:

(4) $$agu_{ck}^{t}=\sum_{j}\frac{au_{kj}^{t}*t_{jc}^{t}}{1-C_{j}^{t}}\;(c=1,\;2;\;j=1,\;2\ldots )$$

where $au_{kj}^{t}$ represents the per capita consumption of the *j*th kind of food by an urban person of gender *k* with the actual diet in year *t*. $C_{j}^{t}$ is the rate of wastage of the *j*th kind of food in the consumption stage in year *t*. $agu_{ck}^{t}$ is the gross grain demand that includes food wasted in actual consumption.

We next consider how this gross consumption of grain *c* would change over time with growth in per capita consumption and accounting for pandemic effects. The per capita grain demand in year *t* + 1 of an urban person of gender *k* ($agu_{ck}^{t+1})$ is given by:

(5) $$agu_{ck}^{t+1}=agu_{ck}^{t}\left(1+u\sigma_{c}^{t}+urgd_{c}^{t}\right)$$

where $urgd_{c}^{t}$ is the average annual growth rate of $gd_{c}^{t}$ in urban areas during the normal years without COVID-19, and $u\sigma_{c}^{t}$ is the adjustment to the growth rate for the impacts of COVID-19.

Equation (6) gives the gross demand for grain type *c* of a rural person of gender *k* in year *t* ($agr_{ck}^{t})$, where $ar_{kj}^{t}$ represents the actual per capita demand for the *j*th kind of food by a rural person The rate of food wastage in the consumption stage was used as the national average level.

(6) $$agr_{ck}^{t}=\sum_{j}\frac{ar_{kj}^{t}*t_{jc}^{t}}{1-C_{j}^{t}}\;(c=1,\;2;\;j=1,\;2\ldots )$$

(7) $$agr_{ck}^{t+1}=agr_{ck}^{t}\left(1+r\sigma_{c}^{t}+rrgd_{c}^{t}\right)$$

the impacts of COVID-19 on the annual growth rate of rural grain demand is represented by $r\sigma_{c}^{t}$, and the average annual growth rate of $gd_{c}^{t}$ in rural areas in normal years is $rrgd_{c}^{t}$.

Adding over the two types of grain gives the food grain demand on average by a person of gender *k* in urban and rural areas in year *t*, $afgu_{k}^{t}$ and $afgr_{k}^{t}$, respectively.

(8) $$afgu_{k}^{t}=\sum_{c=1}^{2}agu_{ck}^{t}$$

(9) $$afgr_{k}^{t}=\sum_{c=1}^{2}agr_{ck}^{t}$$

We next set up the model for S2 based on the dietary guidelines in DGCR 2022. These guidelines are given for three levels of food consumption–low, medium, and high–which we denote by *d* = {1, 2, 3}. We first define the per capita daily demand for grain *c* by a person in the 15–64 age group in year *t* under the guidelines. This demand for grain *c* due to food type *j* in grams, at level *d* ($G_{cdj}^{t})$, is given by the recommended daily consumption of food type *j*, $L_{dj}$, transferred to grain *c* equivalents, and adjusted for food wastage:

(10) $$G_{cdj}^{t}=\frac{L_{dj}*t_{jc}^{t}}{1-C_{j}^{t}}(c=1,\;2,\;3;\;d=1,\;2,\;3;\;j=1,\;2\;\cdots )$$

The annual demand for grain *c* per person in the 15–64 age group, under the guidelines at level *d*, in kilograms ($AG_{cd}^{t})$, is the sum over the food types and annualizing:

(11) $$AG_{cd}^{t}=\underset{j}{\sum }G_{cdj}^{t}*365/1000(c=1,\;2,\;3;\;d=1,\;2,\;3;\;j=1,\;2\;\cdots )$$

$AG_{c3}^{t}$ is thus the food grain demand for the *d* = high level in the guidelines. The average food consumption in age group 15–64 is the highest among the three age groups in our survey [35], and a male consumes more food than a female on average [43]. Following [44], we set the guideline grain *c* demand by a male (*k* = 1) in the standard age group (15–64), in both urban ($su_{c1}^{t}$) and rural areas ($sr_{c1}^{t}$), equal to this high level (*d* = 3) in Equation (12). The guideline demands for a standard age female (*k* = 2) in urban and rural areas are then expressed as a coefficient, fm, multiplied by the male demands in (13) and (14), respectively:

(12) $$su_{c1}^{t}=sr_{c1}^{t}=AG_{cd}^{t}(d=\;3)$$

(13) $$su_{c2}^{t}=fm*su_{c1}^{t}$$

(14) $$sr_{c2}^{t}=fm*sr_{c1}^{t}$$

To summarize so far, in Step 2, we incorporated food waste and COVID-19 factors through the parameters $C_{j}^{t}$ and $u\sigma_{c}^{t}$. Equations (4)–(9) correspond to S1, while Equations (10)–(14) are for S2 where diets change towards the guidelines.

*Step 3. Calculating annual GD*

We next define the national food grain demand in S1, $fgd_{1}^{t}$. This is given by the sum over urban and rural demands; urban demand is given by the food grain demand per standard person in the urban area ($afgu_{k}^{t}$ in Equation (8)) multiplied by the standard population and the urbanization rate$\;\left(ur^{t}\right)$_,_ and rural demand is similarly defined:

(15) $$fgd_{1}^{t}=\overset{3}{\underset{i=1}{\sum }}(\sum_{k=1}^{2}(afgu_{k}^{t}*sp_{ik}^{t}*ur^{t}+afgr_{k}^{t}*sp_{ik}^{t}*\left(1-ur^{t}\right)))$$

The total urban standard population is the standard population at age group *i* multiplied by the urbanization rate and summed over *i*. Similarly, the rural standard population is the standard population at age group *i* multiplied by 1 minus the urbanization rate and summed over *i*. The food grain demand in S2 is derived from the diet guideline demands per standard person given in (12)–(14), multiplied by the respective urban and rural standard persons, and summed over the 3 age groups and 2 genders:

(16) $$fgd_{2}^{t}=\sum_{k=1}^{2}\sum_{i=1}^{3}sp_{ik}^{t}*ur^{t}*su_{ck}^{t}+\sum_{k=1}^{2}\sum_{i=1}^{3}sp_{ik}^{t}*\left(1-ur^{t}\right)*\;sr_{ck}^{t}\;(c=3)$$

This equation takes into account the food grain demand of a standard person in an urban area with a balanced diet in year *t* ($su_{ck}^{t}$) and that of a standard person in a rural area ($sr_{ck}^{t}$).

S3 applies the traditional per capita method that does not distinguish between the different age groups to predict food grain demand under the diet guidelines. Let the urban population of gender *k* in year *t* be $up_{k}^{t}\;$and the total population be $p^{t}$. Recall that the per person food grain demand for the 15–64 age group under the guidelines are given as $su_{ck}^{t}$ and $sr_{ck}^{t}$ in Equations (12)–(14). We first defined the per capita food grain demand in year *t* ($pc^{t}$) as the weighted average of the urban and rural demands, where the population weights are given by $up_{k}^{t}/p^{t}$ and $rp_{k}^{t}/p^{t}$, and summed over the genders:

(17) $$pc^{t}=\sum_{k=1}^{2}su_{ck}^{t}*\frac{up_{k}^{t}}{p^{t}}+\sum_{k=1}^{2}sr_{ck}^{t}*\frac{rp_{k}^{t}}{p^{t}}\;(c=3)$$

The total food grain demand in S3 ($fgd_{3}^{t}$) is then this per capita demand multiplied by the total population:

(18) $$fgd_{3}^{t}=pc^{t}*p^{t}$$

Let lrfd be the lower bound of the ratio of the rest of grain demand (i.e., excepting food grain) to total GD, and urfd be the upper bound ratio. The setting of these bounds is described later in Section 3.1. For each scenario *s*, the upper bound of the total grain demand (food grain plus the rest), $ugd_{s}^{t}$, and the lower bound, $lgd_{s}^{t}$, are then given by:

(19) $$ugd_{s}^{t}=fgd_{s}^{t}/(1-urfd)\;(s=1,\;2,\;3)$$

(20) $$lgd_{s}^{t}=fgd_{s}^{t}/(1-lrfd)\;(s=1,\;2,\;3)$$

Finally, we calculate the total grain demand in scenario *s* in year *t* ($tgd_{s}^{t}$) as the average of the lower and upper bounds:

(21) $$tgd_{s}^{t}=\left(lgd_{s}^{t}+ugd_{s}^{t})/2\;(s=1,\;2,\;3\right)$$

*Step 4. Differences among GD in the 3 scenarios and the decompositions*

In the final step we compare the scenarios and decompose the changes in total grain demand over time. Recall that S1 uses the historical growth rate of consumption, S2 is the transition to the diet guideline, and S3 ignores the age structure of the population. First, we trace the diet guideline effect by calculating the difference rate between S2 and S1 as:

(22) $$\theta^{t}=\frac{tgd_{2}^{t}}{tgd_{1}^{t}}-1$$

Then we trace the aging effect with the difference between S3 and S2 as:

(23) $${µ}^{t}=\frac{tgd_{3}^{t}}{tgd_{2}^{t}}-1$$

We calculate the total staple food grain demand in S1 ($sfgd_{1}^{t})\;$as the per person demands for staple food grain (*c* = 1) multiplied by the standard population and summed over the age and gender groups:

(24) $$sfgd_{1}^{t}=\overset{3}{\underset{i=1}{\sum }}(\sum_{k=1}^{2}(agu_{1k}^{t}*sp_{ik}^{t}*ur^{t}+agr_{1k}^{t}*sp_{ik}^{t}*\left(1-ur^{t}\right)))$$

The staple food grain demand in S2 ($sfgd_{2}^{t}$) is staple food demand per standard person under diet guidelines multiplied by the standard populations in urban and rural areas and summed over the age groups and genders:

(25) $$sfgd_{2}^{t}=\overset{3}{\underset{i=1}{\sum }}(\overset{2}{\underset{k=1}{\sum }}(sp_{ik}^{t}*su_{1k}^{t}*ur^{t}+sr_{1k}^{t}*sp_{ik}^{t}*\left(1-ur^{t}\right)$$

The feed grain demand in S1 and S2 is the total food grain demand less the staple food grain demand, respectively:

(26) $$ffgd_{1}^{t}=fgd_{1}^{t}-sfgd_{1}^{t}$$

(27) $$ffgd_{2}^{t}=fgd_{2}^{t}-sfgd_{2}^{t}\;$$

Given these demands for the total grain, staple food grain and feed grain, we now defined their differences between S1 and S2, respectively, as:

(28) $$dgd^{t}=tgd_{1}^{t}-tgd_{2}^{t}$$

(29) $$dsfgd^{t}=sfgd_{1}^{t}-sfgd_{2}^{t}$$

(30) $$dffgd^{t}=ffgd_{1}^{t}-ffgd_{2}^{t}$$

The share contributions of staple food grain and feed grain differences, $dsfgd^{t}$ and $dffgd^{t}$, to $dgd^{t}$ are defined as:

(31) $$cs\theta^{t}\;=dsfgd^{t}/dgd^{t}$$

(32) $$cf\theta^{t}\;=dffgd^{t}/dgd^{t}$$

We call our model of grain demand (GD) a multi-factor model since it integrates dietary structure, population size, population age structure, urbanization, food waste, and COVID-19 as the main impacting factors of GD. Unlike previous research, which focused on some of these factors, our model provides a more complete representation of the driving factors. Policymakers and grain companies can adapt this model to develop effective grain policies and market strategies. While the model is not exhaustive, it is multidimensional and integrative, allowing stakeholders to modify and incorporate their specialized knowledge to add to the trends identified in this study. That is, the model can act as a building block to guide future research efforts at a higher level of detail.

## 3. Model Application

### 3.1. Grain Consumption in China

The structure of grain consumption in China has changed significantly even in the last decade. The proportion of feed grain in total grain consumption rose from 38.8% in 2013 to 43.0% in 2019, while the ratio of staple food grain fell from 32.6% in 2013 to 27.9% in 2019. The proportions of seed grain, industrial grain, and other grains were more stable during this period, ranging from 28.5% to 30.3% (Figure 2). Based on the information from Figure 2, we set the upper and lower bounds on non-food grain to be: urfd = 30.3%, lrfd = 28.5%.

China’s population and its structure are the main drivers of GD and they are currently undergoing significant changes, including faster aging and new fertility policies [45,46]. In 2021, China officially relaxed its family planning policy further, supporting couples who wish to have a third child. As of the end of 2022, the total domestic population was 1411.8 million, a decrease of 0.85 million from the previous year. The total birth population in 2022 was 0.96 million, and the birth rate was 6.77‰. The total number of deaths was 10.41 million, and the mortality rate was 7.37‰, and the natural growth rate of the population was −0.60‰. This marks the first negative growth of the population in many years. A total of 15.6% of the population is projected to be older than 65 in 2025 [45,46]. The changing population size and, particularly, the aging of the population have been impacting China’s GD and will continue to have significant impacts. In the model, we adopt the standard person consumption ratios, $spcc_{ic}$, and the population size, $p^{t}$, to reflect these changing trends.

The dietary structure of Chinese residents has undergone significant changes with the country’s economic development [9,35]. The proportion of protein derived from animal food consumed by urban and rural residents rose from 18.9% in 1992 to 35.2% in 2015. In rural areas, the energy supply ratio of carbohydrates fell from 70.1% in 1992 to 55.3% in 2015, while the proportion of protein provided by animal food increased from 12.4% to 31.4% [43]. The dietary structure of rural residents has greatly improved, and the gap between urban and rural residents has narrowed due to rural incomes rising relative to urban incomes [10,47,48].

To promote a balanced diet, the Chinese Nutrition Society has issued a series of Dietary Guidelines for Chinese Residents since 1989, with DGCR 2022 being the most recent fifth version [49]. The guidelines recommend 200–300 g of cereal, 50–100 g of tubers, and other foods per day for a healthy person above 2 years of age; these are changed from the earlier guidelines in DGCR 2016 (see Table 1). We set $L_{1j}$ and $L_{3j}\;$as the lower and upper bounds of the recommended amounts of the food *j*, respectively, and $L_{2j}$ as the average of $L_{1j}$ and $L_{3j}$. Although more people are beginning to pay attention to their diets [14], ref. [43] found that over 50% of residents had seriously unbalanced diets in 2021.

China’s urbanization rate, the proportion of urban resident population in the total population, rose steadily from 17.92% in 1978 to 65.22% in 2022. There are significant differences between the dietary structures of urban and rural residents, as shown in Table 2 and Table 3. By comparing these tables with the balanced diet in Table 1, we find that the diets of both urban and rural residents had gradually approached the recommendations in DGCR 2022. On average, the diets of urban residents have fewer cereals/tubers and more vegetables/fruit than rural diets and are thus closer to the recommendations. However, they both still lack sufficient milk and dairy products, and there is excessive consumption of cereal and meat, which is in line with the finding from [50]. The development experience of high-income Asian countries from the 1970s to the 1990s suggests that the increase in urban resident populations leads to higher consumption of meat and milk, which in turn increases the consumption of feed grain. In our model, we incorporate the parameters for urbanization ($ur^{t}$), populations ($up_{k}^{t}$, $rp_{k}^{t}$), diet structure ($au_{kj}^{t}$, $ar_{kj}^{t}$), and food-to-grain coefficients ($t_{jc}^{t}$) to reflect their impacts on GD.

According to the State Food Administration (reported in [51]), China was wasting around 35% of its grain production annually. Additionally, in 2015, food waste in Chinese restaurants was estimated at 17–18 million tons [52,53]. Reference [54] estimated that waste of cereal, tubers and beans in the consumption stage accounted for 5.0% of their total consumption in China, and waste of meat and poultry was 6.4%. The Ministry of Agriculture and Rural Affairs of the People’s Republic of China estimated that the waste ratios for aquatic products, “eggs, milk and dairy,” soybean, and nuts were 15.9%, 4.4%, 2.0%, and 1.0%, respectively [55]. Despite being a significant factor impacting GD, food waste in the consumption stage was often ignored due to poor data. Many have argued that food waste should be included in prediction of future GD [52,56,57]. In our Equations (4), (6) and (10), we account for this waste in consumption stage through the parameter $C_{j}^{t}$.

The global spread of COVID-19 disrupted agricultural production and consumption worldwide, with its impact on China’s grain security expected to persist for 3–5 years, particularly due to its high dependence on imports of agricultural products such as soybeans [58]. Per capita consumption of rice, wheat, and other grains was expected to decline by 1.38%, 1.20%, and 1.33% in 2022 due to strict lockdown measures and the sudden closure of restaurants, local markets, and caterers [59]. In 2020, with the impacts of COVID-19, the annual growth rate of staple food grain consumption was −0.99% [60]. Ref. [61] believed that staple food grain consumption is relatively inelastic and unlikely to fluctuate significantly over the year. During the early stages of the epidemic, some residents and traders hoarded food, which temporarily drove fluctuations in grain market demand but did not alter the annual trend of grain consumption. We accounted for the impacts of COVID-19 using the parameters $u\sigma_{c}^{t}$ and $r\sigma_{c}^{t}$ in Equations (5) and (7), respectively.

### 3.2. Data Source and Parameter Determination

The actual consumption of food types, $f_{ij}^{t}$ (Equation (1)) is derived from a nationwide survey by UCAS in 2017 as reported in [42] (see Table S3). In the survey, 6264 residents of different ages and genders were randomly sampled from the 31 provinces, and the food they consumed for three consecutive days (including working days and weekends) was recorded. The survey took into account the food preferences and ethnic composition of the population across different age groups and regions; factors that are crucial in filling the gaps in available data on food consumption by age groups. Based on the survey data and using Equation (1), we calculated the two types of grain demand, $gd_{ic}^{t}$, for three age groups (0–14, 15–64, 65+) (see Table S4). We then calculated the standard person consumption ratios, $spcc_{ic}$ using Equation (2) (see Table S5).

The diet guidelines, $L_{dj}$, was obtained from the [49] (Table 4). There was a wide range of values for food-to-grain coefficients, $t_{jc}^{t}$, as shown in Table S2, and we calculated values for them using data from [62]. A key factor impacting $t_{jc}^{t}$ is the scale of operation, and Table S6 gives the distribution of sizes of livestock operations. In the Supplementary Materials, we describe how we calculated $t_{jc}^{t}$ based on the scale of operations with Equations (s1)–(s4). Taking improvements in technology into account, the calculated transfer coefficients are given in the last row of Table S2. Our coefficients fall between the minimum and maximum values in the literature noted there. Our final value of $t_{jc}^{t}$ for all food types is reported in Table 4.

The population, $p_{ik}^{t}$, for 2023 to 2025 is forecasted using the method in [45,46] based on the latest population data in 2022, and the results are shown in Table S7. On August 1, 2022, the Chinese government projected that the total population would decline during the 14th Five-Year Plan period (2021–2025). Here we consider the population to have peaked at 1412.60 million in 2021, in line with [63,64]. Most of the previous projections of China’s GD assumed that the population would peak around 2030 [38,55], and this is a source of difference from our projections. We set the female to male consumption ratio, fm = 0.85 according to [43]. We have updated the value of the food waste ratio at the consumption stage, $C_{j}^{t}$, based on the latest literature, including [38,54,55]. Sources of data for key parameters are listed in Table 5, with their values provided in Tables S2–S9.

In 2022, the urbanization rate, $ur^{t}$, reached 65.22%, 0.50 percentage points higher than the 2021. According to the 14th Five-Year Plan (2021–2025) [65], the urbanization rate of China’s permanent population was expected to reach at least 65% by 2025. Jiangsu Province had set a goal of achieving an urbanization rate of 75% by 2025, while Shandong Province aimed for 68%. Using the projections in [65] and taking into account the impacts of COVID-19, we set $ur^{t}$ to be 66.8% in 2023, 67.7% in 2024, and 68.6% in 2025.

The adjustment for COVID-19, $u\sigma_{c}^{t}$, is obtained from three sources—[38,55,60]. The impacts of COVID-19 on grain consumption are consistent across all three sources. For the gap between urban and rural grain consumption per capita in China, [66] found that it decreased from 82 kg per year in 1993 to 51 kg per year in 2009. Ref. [67] estimated the gap to be around 64.6 kg in 2012 and suggested that it would continue to narrow. In this paper, the gap between urban and rural grain consumption of males, $afgu_{1}^{t}$ and $afgr_{1}^{t}$, is extrapolated from the historical trend, the projected values are given in Table S9, the gap narrowing from 45.7 kg in 2017 to 40.6 kg in 2025. The gap in female grain consumption had a similar trend. These results are in line with those in [66,67].

Our data is primarily from the China Statistical Yearbook and the survey conducted by UCAS in 2017. The Statistical Yearbook’s national data excludes Hong Kong, Macau Special Administrative Regions, and Taiwan region, except for administrative divisions, forest resources, and special notes. The UCAS’ survey in 2017 also covered 31 provinces (autonomous regions, and municipalities) in China as well. Hence, the geographic scope of our study is limited to 31 provinces (autonomous regions, and municipalities), excluding the Hong Kong Special Administrative Region, Macau Special Administrative Region, and Taiwan region.

### 3.3. Correlation Test

Our model is a complex mechanistic one, as discussed in the introduction. Researchers using time-series, phenomenological, models may wish to consider the factors that we include. To provide an idea of the possible time-series relationships among our factors—dietary structure, population age structure, urbanization, food waste, and the impacts of COVID-19—we conduct correlation tests. COVID-19 suddenly occurred around 2020 across the world, and the $u\sigma_{c}^{t}$ factor can be regarded as unrelated to the other factors. At the household level, food waste is the outcome of multiple behaviors, including inefficient household food management, confusing expiration date labels, and over-purchasing [57,68,69]. We have not yet found research that shows a strong correlation between food waste and dietary structure, age structure, or urbanization. Unfortunately, we do not have sufficient data to make such a correlation analysis, which is a topic for future research.

With the available data, we made a KMO(Kaiser–Meyer–Olkin)test for the correlation among dietary structure, age structure and urbanization. We use the ratio of meat consumption to total per capita food consumption to reflect dietary structure; using the data for urban residents in Table 2 ($uds^{t})\;$and the data for rural residents ($rds^{t})$ in Table 3. We use the proportion of the population aged 65+ ($uds^{t}$) to indicate age structure. The data of $ur^{t}$, $agr^{t},uds^{t}$ and $rds^{t}$ are listed in Table S10. The KMO value of $rds^{t}$, $agr^{t}$ and $ur^{t}$ is 0.491 (Table S11), the KMO value of $uds^{t}$, $agr^{t}$ and $ur^{t}$ is 0.510 (Table S12), indicating weak correlations among dietary structure, age structure, and urbanization. Therefore, they can be included simultaneously in phenomenological models. Food prices are not explicitly incorporated in our model, [10] and [40] showed that dietary preferences are mostly determined by food prices; that is, dietary structure reflects the price mechanism. Some research considered residents’ income in GD prediction and the income effect on dietary preference is similarly included in our dietary structure variable. The income effect on the level of total food consumption is small in the short run.

## 4. Results

### 4.1. The Projected Grain Demand (GD)

**Figure 4:**
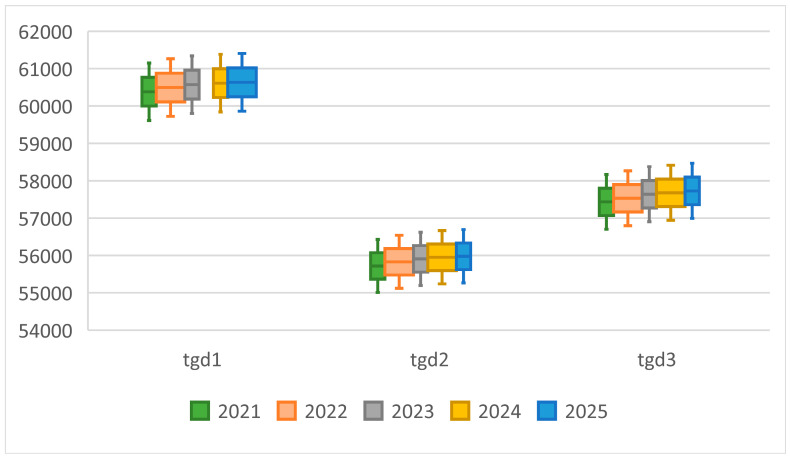
The calculated GD, $tgd_{s}^{t}$**,** in the three scenarios (unit: million tons).

**Figure 5:**
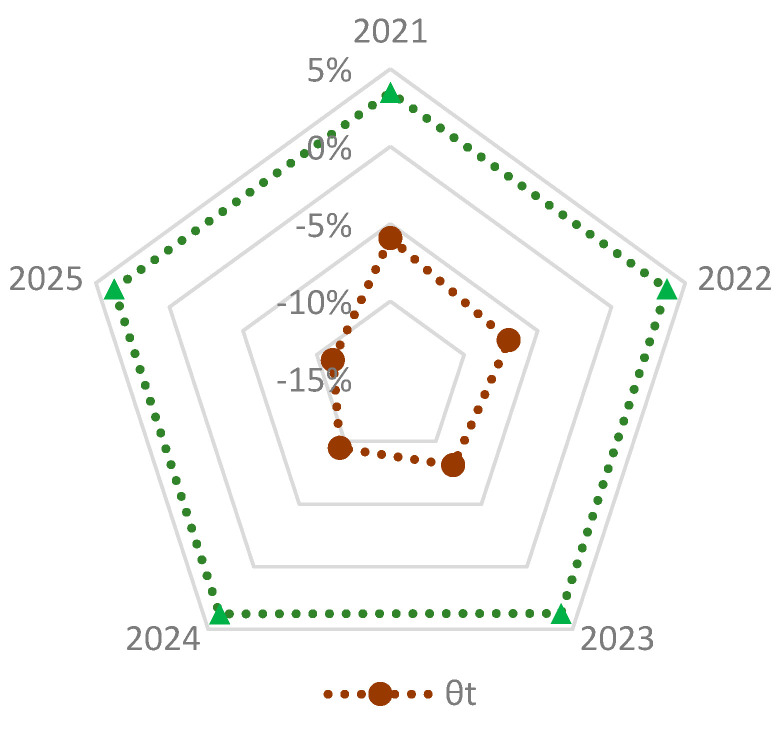
The dietary structure change effect $\theta^{t}$ and aging population effect ${µ}^{t}$ on GD.

Figure 4 reports our projections of the total grain demand ($tgd_{s}^{t}$) during 2022–2025 for the three scenarios, $tgd_{1}^{t}$ is the highest, followed by $tgd_{3}^{t}$ and $tgd_{2}^{t}$. In 2025, the demand for S1, S2 and S3 will be 658.16, 585.06, and 607.09 million tons, respectively. Considering the age structure of the population (S2), this would reduce demand by 21.99 million tons on average during 2022–2025 compared to simple per capita formulas in S3. The difference rate between S2 and S3, ${µ}^{t}$ (Equation (23)) fluctuates a little around 3.76% as shown in Figure 5. The population starts to fall in 2022, and the proportion of the population aged 65+ rises steadily. In 2022, the proportion was 14.9%. If we ignore the age structure of the population, we may overestimate annual GD by 3.8% during 2022–2025.

Although aging and a falling population would reduce GD, changes in urbanization would raise it. In S1, annual GD would rise by 0.9% in 2022, and by 1.2%, 1.5%, 1.8% in 2023, 2024, and 2025, respectively. In S2 the diet guidelines are achieved and Figure 4 shows how GD there would be smaller than in S1; the difference between them, $dgd^{t}$ (Equation (28)), would increase over time, from 43.82 million tons in 2022 to 73.10 million tons in 2025. Accordingly, the difference rate, $\theta^{t}$, would increase in absolute terms from 5.9% in 2022 to 11.1% in 2025 (Figure 5). That is, if Chinese residents fully adopt the DGCR 2022 guidelines, GD would be lower by about 73.10 million tons in 2025, equivalent to 10.7% of China’s grain production in 2021 and about 2.5% of the world grain production in 2020. The results indicate that promoting the diet guidelines more widely would generate a double dividend—a healthier population and a reduction of about 43.82–73.10 million tons of China’s GD annually during 2022–2025, which, in turn, would reduce the pressure on scarce water and land.

**Figure 6:**
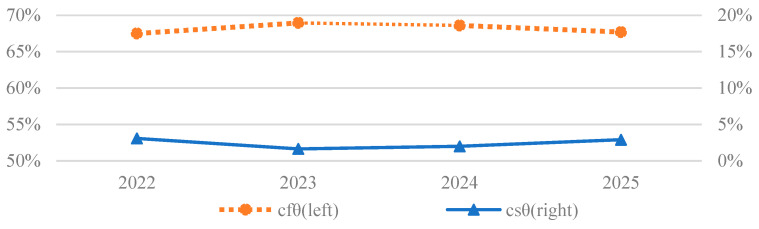
The contribution of the changes in feed grain ($cf\theta^{t})$ and staple food grain$\;(cs\theta^{t})$ to the GD difference between S1 and S2.

Table 6 shows the staple food grain and feed grain composition of projected GD in S1 and S2. In 2022, staple food grain, $sfgd_{1}^{t}$, would account for 27.8% of $tgd_{1}^{t}$, but would fall to 26.7% by 2025. Meanwhile, the feed grain, $ffgd_{1}^{t}$, would comprise 42.8% of $tgd_{1}^{t}$ in 2022 and rise to 43.9% by 2025. As a result of changes in dietary structure, the change in feed grain in S1, $dffgd^{t}$, would be 29.58–49.48 million tons and the change in staple food grain, $dsfgd^{t}$, would be 0.85–2.12 million tons during 2022–2025. In S2, feed grain would average about 40.9% of $tgd_{2}^{t}\;$and staple food grain would average about 29.7% during 2022–2025. Figure 6 shows that the changes in feed grain contributed to 67.5–68.9% of the change in GD, and changes in staple food grain demand contributed only 1.6%–3.1% during 2022–2025.

We have not come across any other research of the effect of using the dietary guidelines in DGCR 2022. Our results showed that GD in S1 would be (7.5%, 12.5%) higher than S2 which follows the guidelines. This shows the significant differences between the recommended diet and the actual diet. Although the components of animal food were not suggested in DGCR 2022, the total amount of animal food consumption suggested in DGCR 2022 is the same as that in DGCR 2016 (Table 1). We assumed that the composition of animal food in DGCR 2022 would be the same as that in DGCR 2016. In 2019, the per capita milk and dairy products consumption of both urban and rural residents in China was less than the minimum volume recommended by DGCR 2022, 254.2 g/day for urban residents and 280.0 g/day for rural residents, respectively. Their consumption of meat and poultry exceeded the maximum values suggested by DGCR 202,234.8 g/day and 20.1 g/day, respectively. As Chinese residents shift to a more balanced diet, they are likely to consume less livestock and poultry and more milk, with the food-to-grain coefficient, $t_{jc}^{t}$, of milk being about 1/7 that of livestock and poultry. This is the main reason why the GD in S1 is expected to be much higher than that in S2. Furthermore, if people consume more aquatic products instead of livestock and poultry meat in the animal food category, the GD could be smaller than what we predicted.

### 4.2. Sensitivity Analysis

Our prediction relies on several parameters, and we want to know how much they affect GD. We conducted a sensitivity analysis on the food waste rates for various types of food ($C_{j}^{t}$), urbanization rate $\left(ur^{t}\right)$, the percentage of residents who adopt DGCR 2022 and report the results in Table 7. We shocked the parameters by 1 percentage point and calculated the impacts on GD in both S1 and S2.

The results in Table 7 show that the largest impact on GD is the waste rate for cereals in S2 $\left(\Delta tgd_{2}^{t}-\mathrm{cereal}\right)$—when the waste rate of cereal is reduced by 1 percentage point, GD in S2 falls by around 2.04 million tons annually during 2022–2025. In recent years, the waste rate of cereal in the consumption stage has been around 5.0% [38]. Next is the impact of the waste rate for livestock and poultry ($\Delta tgd_{2}^{t}-\mathrm{LP}$) where a 1 percentage point fall in the waste rate of livestock and poultry reduces GD by about 1.36 million tons. This is followed by the impact of $\Delta tgd_{2}^{t}-\mathrm{milk}$, about 1.19 million tons.

Raising the urbanization rate by 1 percentage point ($\Delta tgd_{1}^{t}-\mathrm{URS}$) would raise annual GD in S1 by about 0.76 million tons during 2022–2025. If the percentage of Chinese residents who adopt the balanced diet increases by 1 percentage point, about 0.44–0.73 million tons of GD could be annually reduced during 2022–2025. The results suggest that successfully reducing waste of cereal, livestock and poultry meat, and milk in food consumption operations would greatly reduce the pressure of rising GD. Adopting a balanced diet would both reduce GD and improve health.

## 5. Discussion

We report the projections of GD made by other studies in Table S1, noting whether each study considered the age structure of the population or urbanization. They used various methods, including regressions or estimating consumption demand functions. We see that the only study that considered population age structure, [70] projected GD that was lower than the other studies that ignored it. This is consistent with our comparison of S2 and S3. Next, we consider the effect of projections of population size. Ref. [38] projected that the population would peak at 1441.6 million in 2029 and forecasted GD to be 652.06 million tons in 2025, peaking at 676.2 million tons in 2035. However, we forecasted that the population had already peaked in 2021, with a size 2.0% smaller than that of [38]. In S1 we predicted that the lower and upper bounds of China’s GD ($lgd_{1}^{t}\;$and $ugd_{1}^{t}$) in 2025 would be 649.77 and 666.55 million tons, respectively. Besides having a different peak population, [38] also did not consider the age structure of the population. These are the main reasons why their peak GD (676.2 million tons) is larger than our $ugd_{1}^{t}$ (666.55 million tons) in 2025, the highest value we predicted between 2022–2025.

We recognize some limits to the implementation of our model here. First, due to survey limitations, we were only able to distinguish three age groups (0–14, 15–64, and 65 and over) when calculating the standard person consumption ratios, $spcc_{ic}$. If we had access to more detailed age group data, we would most likely project an even lower GD, i.e., the calculated ratio between S3 and S2, $\mu^{t}$, may have been even larger. Secondly, we considered gender in scenario S1, but in scenario S2, we did not have separate diet recommendations for males and females and were unable to account for a change in gender structure, which may lead to a slightly different GD. Thirdly, we should note that, as discussed in the review of models by [16], they did not identify any of the complex model studies reporting attempts at model verification by discussing AIC or R2 statistics or employing cross-validation approaches due to data limitations; papers using simpler mechanistic models did report some form of model validation. Similarly, our model integrates numerous impact factors with multiple parameters; unfortunately, there is not enough time series data to apply statistical methods to estimate these parameters. For some parameters such as$\;spcc_{ic}$, $C_{j}^{t}$, $t_{jc}^{t}$, it is difficult to find even just one recent year of data. Fortunately, from a qualitative perspective, their short-term fluctuations are not significant. Some recent studies have conducted statistical analysis on the trend changes of some parameters, such as the COVID adjustment ($u\sigma_{c}^{t})$ and food waste ($C_{j}^{t}$). We apply the values of $u\sigma_{c}^{t}\;$and $C_{j}^{t}$. that have been consistently estimated in the literature. The parameters for standard persons and food-grain conversion ($spcc_{ic}$, $t_{jc}^{t})$ had been estimated with long lags and differ significantly across studies and we recalibrated them using the latest data. We assumed that they would remain on their current trend during our short-term prediction period of 2022–2025. This short-term forecasting assumption is used in many previous studies that use phenomenological (time-trend) models, including those that project out to 2050 [16,20]. We endeavored to be transparent with our determination of parameter values and presented the results of the sensitivity analyses, which we hope are sufficient to give a sense of the range of uncertainty.

This study provides some useful practical results for grain policymakers, grain companies, and anyone interested in enhancing grain security. First, our model allows for a more comprehensive understanding of the various factors that contribute to GD, enabling policymakers and officials to better prepare for uncertainties in the future and help target grain supply policy. Better policymaking will benefit both producers and consumers. Second, our results show that adopting the recommended diets would not lead to an increase in China’s GD but would benefit human health and the environment. Our calculation of the drop in GD provides a rigorous basis for promoting the adoption of the dietary guidelines in DGCR 2022. Third, [71] estimated the annual food loss and waste in China were at least 120 million tons, making it a significant concern that cannot be overlooked. Our sensitivity analysis indicates that reducing consumption of food waste, particularly cereal, livestock and poultry, and milk waste, would have the most significant effects on reducing GD. This points towards the need for effective policies to reduce food waste. Fourth, we noted that [72] estimated that global food consumption alone could add nearly 1 °C to warming by 2100, with 75% of this warming driven by high-methane foods such as ruminant meat, dairy, and rice. On the other hand, they also argued that simultaneous improvements in production practices, the universal adoption of healthy diets, and reductions in consumer- and retail-level food waste could avoid over 55% of the anticipated warming. Projections based on our integrated model could estimate the methane emissions and other greenhouse gases from China’s grain consumption and show how these emissions may be reduced by promoting a healthy diet and reducing food waste. Such research would have practical and significant implications. Finally, we calculated that the inclusion of aging effects reduces projected GD by 3.8%. This is equivalent to an annual savings of around 0.3 billion RMB in grain inventory costs. These findings are not only relevant for China but can also serve as a valuable reference for predicting GD in other countries that are facing similar demographic challenges related to aging populations.

## 6. Conclusions

We predict China’s GD in the short term, comprehensively demonstrating how changes in the dietary structure, population size and age structure, food waste, urbanization, and COVID-19 can impact GD. We have four main findings. First, if the change in diets continues at the rate observed during 2013–2019, China’s GD would reach 658.16 million tons in 2025. Secondly, if all residents adopted the balanced diet suggested by DGCR 2022, China’s GD would drop to 585.06 million tons in 2025. This would result in a 5.9–11.1% decrease in annual GD during 2022–2025, with 68% of the decrease due to feed grain demand. For every 1 percentage point increase in residents adopting the balanced diet during 2022–2025, annual GD would fall by 0.44–0.73 million tons over 2022–2025. The results suggest that promoting DGCR 2022 guidelines would have the double dividend of a healthier population and reducing GD in China. Our third finding is that ignoring the age structure of the population could lead to an overprediction of annual GD of about 3.8% during 2022–2025. The fourth finding shows how reducing waste of cereal, livestock and poultry meat, and milk would be an effective alternative to reducing the pressure on GD, offsetting the higher demand due to rising urbanization and higher incomes. The results of the analysis provided potential ways to reduce China’s GDP. The results should be useful for the government to formulate grain supply plans and policies to effectively reduce GD as a contribution towards a balanced diet for the people. These findings highlight the critical need to account for multiple factors simultaneously, especially dietary structure and demographic composition, resulting in a more accurate prediction of GD. Further research is necessary to gain deeper insights into strategies for promoting widespread adoption of balanced diets and effectively curbing food waste. Furthermore, our modeling approach may be used to better link food consumption with methane emissions and other greenhouse gases, elucidating how these emissions can be mitigated through the promotion of healthy dietary practices and the reduction of food waste.

## Figures and Tables

**Table 1:** The suggested per capita daily foods consumption by a person older than 2 years old by DGCR 2022 and DGCR 2016(Unit: g/day).

Items	2016	Items	2022
Cereal, tubers and beans	250–400	Cereal	200–300
		Tubers	50–100
Vegetables	300–500	Vegetables	300–500
Fruits	200–350	Fruits	200–350
Livestock and poultry meat	40–75	Animal food	120–200
Aquatic products	40–75		
Eggs	40–50		
Milk and dairy products	300	Milk and Dairy	300–500
Soybean and nut	25–35	Soybean and Nut	25–35
Oil	25–30	Oil	25–30

**Table 2:** The per capital major food consumption of urban residents in China (Unit: g/day).

Foods	2013	2014	2015	2016	2017	2018	2019	2020
Cereal, tubers and beans	303.0	291.8	278.4	275.3	270.1	270.7	269.9	294.1
Meat	78.1	77.8	79.2	79.5	80.0	85.5	78.6	75.0
Pork	55.9	57.0	56.7	55.9	56.4	62.2	55.6	52.2
Beef	6.0	6.0	6.6	6.8	7.1	7.4	7.9	8.4
Mutton	3.0	3.3	4.1	4.9	4.4	4.1	3.8	3.8
Poultry	22.2	24.9	25.8	27.9	26.6	26.8	31.2	35.6
Aquatic products	38.4	39.5	40.3	40.5	40.5	39.2	45.8	45.6
Eggs	25.8	26.8	28.8	29.3	29.9	29.6	31.5	37.0
Milk and dairy products	46.8	49.6	46.8	45.2	45.2	45.2	45.8	47.5
Oil	29.9	30.1	30.4	30.1	29.3	25.8	25.2	27.1

Data Source: China Statistical Yearbook 2014–2021.

**Table 3:** The per capital major food consumption of rural residents in China (Unit: g/day).

Foods	2013	2014	2015	2016	2017	2018	2019	2020
Cereal, tubers and beans	465.2	435.9	411.5	403.0	396.7	377.8	390.7	424.5
Meat	61.4	61.6	63.3	62.2	64.7	75.3	67.7	58.8
Pork	52.3	52.6	53.4	51.2	53.4	63.0	55.3	46.9
Beef	2.2	2.2	2.2	2.5	2.5	3.0	3.3	3.6
Mutton	1.9	1.9	2.5	3.0	2.7	2.7	2.7	2.7
Poultry	17.0	18.4	19.5	21.6	21.6	21.9	27.4	34.1
Aquatic products	18.1	18.6	19.7	20.5	20.3	21.4	26.3	28.3
Eggs	19.2	19.7	22.7	23.3	24.4	23.0	26.3	32.3
Milk and dairy products	15.6	17.5	17.3	18.1	18.9	18.9	20.0	20.1
Oil	28.2	26.8	27.7	27.9	27.7	27.2	26.9	30.0

Data Source: China Statistical Yearbook 2014–2021. Note: The data released by the National Bureau of Statistics after 2012 showed an obvious change from earlier data because the statistical scope was adjusted. Therefore, we used the statistical data beginning in 2013.

**Table 4:** The calculated food-to-grain coefficients $t_{jc}^{t}$, of the cited food waste ratios at consumption stage$\;C_{j}^{t}$, and diet guidelines in DGCR 2022 $L_{dj}$.

Foods	$t_{jc}^{t}$	$C_{j}^{t}$	$L_{1j}$	$L_{2j}$	$L_{3j}$
		Food Waste Ratio at Consumption Stage	Diet Guidelines in DGCR 2022		
Cereal, tubers and beans-1	1.00	5.0% (Zheng et al., 2022 [38])	250	325	400
Livestock and poultry meat-2	2.65	6.4% (Zhou et al., 2019 [54])	40	58	75
Aquatic products-3	0.90	15.9% (MEWEC, 2021 [55])	40	58	75
Eggs-4	1.66	4.4% (MEWEC, 2021 [55])	40	45	50
Milk and dairy products-5	0.38	2.0% (MEWEC, 2021 [55])	300	400	500
Soybean and nut Oil-6	1.00	1.0% (MEWEC, 2021 [55])	25	30	35

**Table 5:** Sources of data for key parameters.

Parameters	References
$t_{jc}^{t}$	Table S2
$f_{ij}^{t}$	A survey conducted by UCAS in 2017
$rrgd_{c}^{t}$	China Statistical Year Book 2014–2021
$urgd_{c}^{t}$	China Statistical Year Book 2014–2021
$spcc_{ic}$	Table S3
$C_{j}^{t}$	Zheng et al. (2022) [38]; MEWEC (2021) [55] and Zhou et al. (2019) [54]
$p_{ik}^{t}$	China Statistical Yearbook 2014–2021
$p^{t}$	China Statistical Yearbook 2014–2021
$agu_{kj}^{t}$	A survey conducted by UCAS in 2017
$agr_{kj}^{t}$	A survey conducted by UCAS in 2017
$ur^{t}$	Zhang (2021) [65]
fm	0.85 (Chinese Nutrition Society, 2021 [43])
$up_{k}^{t}$	China Statistical Yearbook 2014–2021
$rp_{k}^{t}$	China Statistical Yearbook 2014–2021
$r\sigma_{c}^{t}$	Zheng et al. (2022) [38]; MEWEC (2021) [55] and Zhong et al. (2021) [60]
$u\sigma_{c}^{t}$	Zheng et al. (2022) [38]; MEWEC (2021) [55] and Zhong et al. (2021) [60]
lrfd	FAOSTAT statistical data during 2013–2019
u rfd	FAOSTAT statistical data during 2013–2019

**Table 6:** The components of GD in scenarios S1 and S2 ($tgd_{1}^{t},$ $tgd_{2}^{t})$, and components of the change in GD in the two scenarios ($dffgd^{t},dsfgd^{t})$.

	2022	2023	2024	2025
$sfgd_{1}^{t}/tgd_{1}^{t}$	27.8%	27.4%	27.0%	26.7%
$ffgd_{1}^{t}/tgd_{1}^{t}$	42.8%	43.2%	43.5%	43.9%
$sfgd_{2}^{t}/tgd_{2}^{t}$	29.7%	29.7%	29.7%	29.7%
$ffgd_{2}^{t}/tgd_{2}^{t}$	40.9%	40.9%	40.9%	40.9%
$dffgd^{t}$	2958	3554	4200	4948
$dsfgd^{t}$	135	85	122	212

**Table 7:** The sensitivity analysis of how key parameters affect GD (Unit:10,000 tons).

Index	Meaning of Index	2022	2023	2024	2025
$\Delta tgd_{1}^{t}-URS$	The change of $tgd_{1}^{t}$(S1) when urbanization rate increases by 1 percentage point	75.09	75.53	76.26	76.96
$\Delta tgd_{1}^{t}-DS$	The change of $tgd_{1}^{t}\left(S1\right)\;$when people who adopt DGCR 2022 increase by 1 percentage point	−43.83	−51.55	−61.23	−73.10
$\Delta tgd_{2}^{t}-cereal$	The change of $tgd_{2}^{t}$(S2) when the waste of cereal decreases by 1 percentage point	203.6	203.6	203.5	203.4
$\Delta tgd_{2}^{t}-LP$	The change of $tgd_{2}^{t}\left(S2\right)$ when the waste of livestock and poultry meat decreases by 1 percentage point	135.7	135.8	135.7	135.6
$\Delta tgd_{2}^{t}-milk$	The change of $tgd_{2}^{t}\left(S2\right)$ when the waste of milk decreases by 1 percentage point	118.8	118.8	118.7	118.7
$\Delta tgd_{2}^{t}-eggs$	The change of $tgd_{2}^{t}\left(S2\right)$ when the waste of eggs decreases by 1 percentage point	50.9	50.9	50.9	50.9
$\Delta tgd_{2}^{t}-AP$	The change of $tgd_{2}^{t}\left(S2\right)$ when the waste of aquaculture product decreases by 1 percentage point	50.9	50.9	50.9	50.9

## Data Availability

Data used during the current study are available from the corresponding author.

## Supplementary Materials

The following supporting information can be downloaded at: https://www.mdpi.com/article/10.3390/nu15132877/s1, References [44,59,60,62,66,70,73,74,75,76,77,78,79,80,81,82] are cited in supplementary material.
