# Supplementary material for: Aging Population, Balanced Diet and China’s Grain Demand
Source: Nutrients. 2023 Jun 25;15(13):2877. doi: 10.3390/nu15132877 (PMC10346198; doi:10.3390/nu15132877)
Supplement: Supplementary file 1 [file nutrients-15-02877-s001.zip › nutrients-2442338-supplementary.pdf]

nutrients

MDPI

Aging Population, Balanced Diet and China's Grain Demand

Supplementary Materials

Table S1. Other projections of China's GD

	Methods/assumptions	Predicted year	Population age structure	Urban-rural structure	Predicted China's GD (unit: million tons)
Gao [73]	regression method	2020	No	no	590
Tang and Li [44]	the per capita demand and the trend prediction method	2020	No	no	567
Yuan *et al*. [70]	moving average method and standard person consumption ratio	2020	Yes	yes	480
Wang and Yu [74]	autoregressive integrated moving average -generalized regression neural network model	2020	No	no	741
Zhang and Xu [66]	Almost Ideal Demand System (AIDS) model	2020	No	yes	694
Tang and Li [44]	the per capita demand and the trend prediction method	2030	No	yes	586
Yuan *et al*. [70]	moving average method and standard person consumption ratio	2030	Yes	yes	560

Table S2. Food-to-grain conversion coefficient, $coef_m$, in existing literature and our estimates in this paper

Literature	Research region	Pork	Beef	Mutton	Poultry	Eggs	Milk and dairy products	Aquatic products
Xin *et al*. [75]	China, province	2.7	2.1	2.2	2.3	1.9	0.4	1.0
Chinese Academy of Agricultural Sciences [76]	China	3.0	1.9	1.9	2.15	1.65	0.36	
Ma and Niu [19]	China	3.00	2.00	2.00	2.00	2.00	0.33	1.00
Han *et al*. [77]	China	2.72	1.74	1.55	2.08	1.65	0.55	0.44
Meng [78]	China	3.00	2.00	2.00	2.00		0.30	0.80
Chen [79]	China	3.26			2.42	2.31		0.95

Wang [80]	Province	3.08	3.24	3.19	2.71	1.67	0.36	1.28
Xie *et al*. [81]	China	2.70	2.50	2.92	2.03	1.68	0.27	1.28
Cao *et al*. [82]	China	1.85	0.85	1.03	1.77	1.64	0.37	
Maximum		3.26	3.24	3.19	2.71	2.31	0.55	1.28
Minimum		1.85	0.85	1.03	1.77	1.64	0.27	0.44
Median		3.00	2.00	2.00	2.08	1.68	0.36	1.00
This paper ($coef_m$)	China	2.77	2.15	2.41	2.56	1.66	0.38	0.90

To calculate the food-to-grain conversion coefficients, we first consider the feeding scale effect. We calculated the feed grain consumption coefficients of animal food m at feeding scale *fs* ($\boldsymbol{coef}_{fs,m}$)using equations (s1), where $\boldsymbol{fgrain}_{fs,m}$ is the feed grain consumption to produce animal food m at feeding scale *fs*, $\boldsymbol{p}_{fs,m}$ is the "live weight" of animal food, and $\boldsymbol{sr}_m$ is the slaughter rate of animal food m (the ratio of the carcass weight after slaughter to the live weight). Then we compute the mean conversion coefficient, $\boldsymbol{coef}_m$ as the weighted average in equation (s2).$\boldsymbol{w}_{fs}$ is the proportion of different feeding scale *fs*. *fs*=1 corresponds to scatter feeding (<30), *fs*=2 corresponds to small-scale feeding (30-100), etc. as given in Table S6.

$$\boldsymbol{coef}_{fs,m} = \frac{\boldsymbol{fgrain}_{fs,m}}{\boldsymbol{p}_{fs,m} * \boldsymbol{sr}_m} \quad \text{(s1)}$$

$$\boldsymbol{coef}_m = \sum_{fs} \boldsymbol{w}_{fs} * \boldsymbol{coef}_{fs,m} \quad (fs=1, 2, 3, 4) \quad \text{(s2)}$$

The data for $\boldsymbol{fgrain}_{fs,m}$ and $\boldsymbol{p}_{fs,m}$ are from the National Agricultural Product Revenue Compilation 2021 [62], the slaughter rates ($\boldsymbol{sr}_m$) of pig, cattle, mutton and broiler chicken were set at 0.7, 0.55, 0.47 and 0.7, respectively, using information in [75]. For laying chicken and cows that do not need to be slaughtered, we set the slaughter rate to be 1. The values of $\boldsymbol{w}_{fs}$ are in parenthesis in Table S6. In UCAS survey data, pork, beef, mutton and poultry were integrated into one category named livestock and poultry (j=2) in Table 7. We estimate $\boldsymbol{t}_{2c}^{t}$using equation (s3), as the weighted average value of $\boldsymbol{coef}_m$of four kinds of meat, using the consumption of rural and urban residents as weights, and averaging over 2017-2019.

$$\boldsymbol{t}_{2c}^{t} = \left(\frac{1}{3} * \sum_{t=2017}^{2019} \sum_m \boldsymbol{coef}_m * \boldsymbol{rlp}_m^t + \frac{1}{3} * \sum_{t=2017}^{2019} \sum_m \boldsymbol{coef}_m * \boldsymbol{ulp}_m^t\right)/2 \quad \text{(s3)}$$

(when $m \in$pork, beef, mutton, poultry)

$$\boldsymbol{t}_{jc}^{t} = \boldsymbol{coef}_m \quad \text{(when } j \text{ and } m \neq \text{pork, beef, mutton, poultry)} \quad \text{(s4)}$$

In (s3), $\boldsymbol{rlp}_m^t$and $\boldsymbol{ulp}_m^t$ are the consumption proportions of pork, beef, mutton and poultry (of their sum by weight) of rural and urban residents, respectively, in year t. The categories of aquatic products, eggs, milk and dairy in the UCAS survey are the same as those of classification in Table S2. We simply set $\boldsymbol{t}_{jc}^{t}$=$\boldsymbol{coef}_m$ for types of food that are not livestock and poultry meat.

Table S3. The surveyed value of average food consumption, $\boldsymbol{f}_{ij}^{t}$, in China in 2017 (Unit: g/ per capita per day)

Age groups	Cereal, tubers and beans	Eggs	Livestock and poultry products	Aquatic products	Milk and dairy products
0-14	241.9	48.4	96.3	29.4	65.1
15-64	392.0	59.1	150.4	45.7	20.5
>=65	363.2	51.6	107.9	32.5	14.2

Table S4. The estimated grain demand, gd_{ic}^{t}, in China in 2017 based on the survey of UCAS (Unit: g/ capita/ day)

Age groups	Staple food grain	Feed grain	Food grain
0-14	241.9	386.6	628.5
15-64	392.0	545.6	937.6
>=65	363.2	406.1	769.3

Table S5. Estimated standard person consumption ratio, $spcc_{ic}$, in 2017

Age groups	Staple food grain	Feed grain	Food grain
0-14	0.62	0.71	0.67
15-64	1.00	1.00	1.00
>=65	0.93	0.75	0.82

Table S6. Feeding scale structure of livestock and poultry in China

Scale classification	Feeding Scale structure			
(Proportion)	Scatter-feeding (*fs*=1)	Small-scale feeding (*fs*=2)	Medium-scale feeding (*fs*=3)	Extensive-scale feeding (*fs*=4)
Pig	<30<br>(0.566)	30-100<br>(0.410)	100-1000<br>(0.022)	>1000<br>(0.003)
Broiler chicken	<300<br>(0.148)	300-1000<br>(0.345)	1000-10000<br>(0.501)	>10000<br>(0.007)
Laying chicken	<300<br>(0.580)	300-1000<br>(0.393)	1000-10000<br>(0.024)	>10000<br>(0.004)
Cow	<10<br>(0.910)	10-50<br>(0.073)	50-500<br>(0.015)	>500<br>(0.003)
Cattle	<50<br>(0.990)		>50<br>(0.010)	
Lamb	<100<br>(0.964)		>100<br>(0.036)	

Data source: Wind.

Table S7. The projected population in China for 2023-2025 (Unit:10000 persons)

Year	up_1^t	up_2^t	rp_1^t	rp_2^t	p^t
2023	48019.4	46280.6	23866.0	23001.7	141167.6
2024	48357.5	47191.0	23071.6	22515.1	141134.9
2025	48976.7	47795.3	22417.9	21877.2	141066.9

Table S8. Annual growth rate of grain demand and COVID-19 adjustment rates; rural and urban ($\boldsymbol{rrgd_c^t}, \boldsymbol{r\sigma_c^t}, \boldsymbol{urgd_c^t}, \boldsymbol{u\sigma_c^t}$)

	$\boldsymbol{rrgd_1^t}$	$\boldsymbol{rrgd_2^t}$	$\boldsymbol{r\sigma_1^t}$	$\boldsymbol{r\sigma_2^t}$
Data source	the average growth speed during 2015-2019		Lin *et al*.[59]; Zhong *et al*.[60]	
2022	0.62%	3.29%	-1.00%	-1.50%
2023	0.62%	3.29%	-1.10%	-1.30%
2024	0.62%	3.29%	-0.60%	-1.10%
2025	0.62%	3.29%	-0.30%	-0.80%
	$\boldsymbol{urgd_1^t}$	$\boldsymbol{urgd_2^t}$	$\boldsymbol{u\sigma_1^t}$	$\boldsymbol{u\sigma_2^t}$
Data source	the average growth speed during 2015-2019		Lin *et al*.[59]; Zhong *et al*.[60]	
2022	1.10%	1.9%	-1.20%	-1.40%
2023	1.10%	1.9%	-1.30%	-1.10%
2024	1.10%	1.9%	-0.80%	-0.90%
2025	1.10%	1.9%	-0.50%	-0.50%

Table S9. Estimated food grain demand per person; urban and rural ($\boldsymbol{afgu_k^t}$, $\boldsymbol{afgr_k^t}$) k=1 is male, k=2 is female (Unit: kg/year)

	$\boldsymbol{afgu_1^t}$	$\boldsymbol{afgu_2^t}$	$\boldsymbol{afgr_1^t}$	$\boldsymbol{afgr_2^t}$
2012 (Xin *et al* [18])	358.1	358.1	293.5	293.5
2017	372.7	316.8	327.0	271.1
2018	376.6	320.1	332.1	275.3
2019	378.9	322.0	335.8	278.4
2020	381.8	324.5	340.0	281.9
2021E	382.5	325.1	342.4	283.8
2022E	386.3	328.3	345.8	286.7
2023E	390.5	331.9	349.8	290.0
2024E	395.9	336.5	354.9	294.2
2025E	402.7	342.3	361.3	299.5
	$\boldsymbol{su_1^t}$	$\boldsymbol{su_2^t}$	$\boldsymbol{sr_1^t}$	$\boldsymbol{sr_2^t}$
2022-2025E	345.1	293.7	345.1	293.7
	$\boldsymbol{afgu_1^t}$	$\boldsymbol{afgu_2^t}$	$\boldsymbol{afgr_1^t}$	$\boldsymbol{afgr_2^t}$
2022-2025E	303.4	303.4	303.4	303.4

Table S10. The data of $\boldsymbol{ur^t}$, $\boldsymbol{agr^t}, \boldsymbol{uds^t}$and $\boldsymbol{rds^t}$

	2013	2014	2015	2016	2017	2018	2019	2020
$\boldsymbol{ur^t}$	0.5373	0.5477	0.561	0.5735	0.5852	0.5958	0.606	0.6399
$\boldsymbol{agr^t}$	0.0970	0.1010	0.1050	0.1080	0.1140	0.1190	0.1260	0.1350
$\boldsymbol{uds^t}$	0.1435	0.1439	0.1495	0.1506	0.1534	0.1635	0.1489	0.1335
$\boldsymbol{rds^t}$	0.0983	0.1029	0.1088	0.1079	0.1127	0.1332	0.1157	0.0936

Data Source: China Statistical Yearbook 2014-2021

Table S11. KMO-and-Bartlett's-Test results of rds^t, $agr^t$and ur^t during 2013-2020

KMO measure of sampling adequacy		0.491
Bartlett Test of Sphericity	chi – squared approximation	21.901
	degree of freedom	3
	significance	0.000

Table S12. KMO-and-Bartlett's-Test results of uds^t, $agr^t$and ur^t during 2013-2020

KMO measure of sampling adequacy		0.510
Bartlett Test of Sphericity	chi – squared approximation	21.751
	degree of freedom	3
	significance	0.000
